# Amitriptyline at low-dose and titrated for irritable bowel syndrome as second-line treatment (The ATLANTIS trial): protocol for a randomised double-blind placebo-controlled trial in primary care

**DOI:** 10.1186/s13063-022-06492-6

**Published:** 2022-07-08

**Authors:** Sarah L. Alderson, Alexandra Wright-Hughes, Alexander C. Ford, Amanda Farrin, Suzanne Hartley, Catherine Fernandez, Christopher Taylor, Pei Loo Ow, Emma Teasdale, Daniel Howdon, Elspeth Guthrie, Robbie Foy, Matthew J. Ridd, Felicity L. Bishop, Delia Muir, Matthew Chaddock, Amy Herbert, Deborah Cooper, Ruth Gibbins, Sonia Newman, Heather Cook, Roberta Longo, Hazel Everitt

**Affiliations:** 1 School of Medicine, Leeds Institute of Health Sciences, University of Leeds, University of Leeds, Room 10.39, Worsley Building, Clarendon Way, Leeds, LS9 9LU UK; 2 Clinical Trial Research Unit, Leeds Institute of Clinical Trials Research, School of Medicine, University of Leeds, Leeds, UK; 3 Leeds Gastroenterology Institute, St James’s University Hospital, Leeds, UK; 4 Leeds Institute of Medical Research at St. James’s, University of Leeds, Leeds, UK; 5 Centre for Clinical and Community Applications of Health Psychology, School of Psychology, University of Southampton, Southampton, UK; 6 Population Health Sciences, Bristol Medical School, University of Bristol, Bristol, UK; 7 Let’s Cure IBS, Sheffield, UK; 8 Primary Care Research Centre, Faculty of Medicine, University of Southampton, Southampton, UK; 9 Exeter Clinical Trials Unit, University of Exeter, Exeter, UK

**Keywords:** Irritable bowel syndrome, Amitriptyline, Primary care, Double-blind, Placebo, Randomised controlled trial

## Abstract

**Background:**

Irritable bowel syndrome (IBS) is a common functional bowel disorder that has a considerable impact on patient quality of life and substantial societal and health care resource costs. Current treatments are often ineffective. Tricyclic antidepressants have shown promise in secondary care populations but their effectiveness in a primary care setting remains unclear.

**Methods:**

ATLANTIS is a randomised, multi-centre, parallel-group, two-arm, double-blind, placebo-controlled trial of low-dose amitriptyline as a second-line treatment for IBS in primary care. Participants will be invited by letter, or recruited opportunistically, from general practices in three regions of England (West Yorkshire, Wessex, and West of England) and screened for eligibility. A total of 518 adult patients with IBS, who are symptomatic despite first-line therapies, will be randomised 1:1 to amitriptyline or identical placebo for 6 months. Treatment will commence at a dose of 10 mg (or one placebo tablet) daily at night, with dose titration up to a maximum of 30 mg at night, depending on side effects and response to treatment. Participant-reported assessments will be conducted at baseline and 3, 6, and 12 months post-randomisation. The primary objective is to determine the effectiveness of amitriptyline, compared with placebo, in improving participant-reported global symptoms of IBS at 6 months (using the IBS Severity Scoring System). Secondary outcomes include relief of IBS symptoms, effect on IBS-associated somatic symptoms (Patient Health Questionnaire-12), anxiety and depression (Hospital Anxiety and Depression Scale), ability to work and participate in other activities (Work and Social Adjustment Scale), acceptability and tolerability of treatment, self-reported health care use, health-related quality of life (EQ-5D-3L), and cost-effectiveness. A nested, qualitative study will explore patient and general practitioner experiences of treatments and trial participation, including acceptability, adherence, unanticipated effects, and implications for wider use of amitriptyline for IBS in primary care.

**Discussion:**

Determining the clinical and cost-effectiveness of low-dose amitriptyline as a second-line treatment for IBS in primary care will provide robust evidence to inform management decisions.

**Trial registration:**

ISRCTN ISRCTN48075063 . Registered on 7th June 2019.

**Supplementary Information:**

The online version contains supplementary material available at 10.1186/s13063-022-06492-6.

## Introduction

### Background and rationale

Irritable bowel syndrome (IBS) is a common, chronic disorder of gut-brain interaction. Symptoms, which include abdominal pain and altered stool form or frequency, range from mild to severe, but are often recurrent and unpredictable [1]. The prevalence of IBS in the community is between 5 and 10% [2], and the condition accounts for > 3% of all consultations in primary care [3]. Impact on quality of life and social functioning can be substantial and comparable with organic bowel disorders, such as Crohn’s disease [4, 5]. The pathophysiology of IBS is incompletely understood, there is no cure, and medical management can be challenging and unsatisfactory. Treatment is usually focused on improving symptoms and quality of life. Current first-line therapies, as recommended by the National Institute for Health and Care Excellence (NICE), include a clear explanation of the condition and information sharing on self-management, dietary and lifestyle advice, soluble fibre, laxatives, and antispasmodic or anti-diarrhoeal drugs [6]. However, if these measures are ineffective, general practitioners (GPs) are often left with few effective treatment options and patients with ongoing symptoms may be referred for a specialist opinion in secondary care [7]. New therapies for IBS continue to be developed; however, they are usually modest in terms of their efficacy and are expensive and, in recent years, several have been withdrawn due to serious concerns about side effects [8, 9].

The NICE IBS guideline states that GPs should “consider” low-dose tricyclic antidepressants (TCAs) for their analgesic effect as second-line treatment for IBS, if first-line therapies have not helped [6]. A possible mode of action is through the pain-modifying properties of low-dose TCAs [10–13] and their action on gastrointestinal motility [14, 15] rather than any effect on mood. However, survey results indicate less than 10% of GPs prescribe TCA for IBS often, and only 50% believe they are effective [16]. Although systematic reviews and meta-analyses suggest that low-dose TCAs may be effective in the treatment of IBS [17–19], important limitations exist in the current evidence. In the most recent review of 12 trials and 787 patients [18], almost all studies were conducted in specialist settings, and the duration of follow-up was limited to 12 weeks, which is insufficient for a chronic fluctuating condition. It is unclear whether these results would translate to a primary care population, and whether there would be an impact on resource use, quality of life, and social functioning. The NICE guideline highlighted the need for a randomised controlled trial (RCT) of TCAs in primary care [6]. Amitriptyline was chosen for this trial as it has shown promise in two small previous trials [20, 21]; is an established, inexpensive drug, which GPs prescribe commonly for other conditions; and has a well-characterised safety profile [22].

### Aim and objectives

The AmitripTyline at Low-dose ANd Titrated for Irritable bowel syndrome as Second-line treatment (ATLANTIS) trial aims to determine the clinical and cost-effectiveness of amitriptyline compared with placebo in patients with IBS in primary care.

#### Primary objective

To determine the effect of amitriptyline on global symptoms of IBS as measured by the IBS Severity Scoring System (IBS-SSS) [23], 6 months after randomisation.

#### Secondary objectives

To assess the effect of amitriptyline on:

1. Global symptoms of IBS, as measured by the IBS-SSS at 3 and 12 months
2. The proportion of participants with relief of IBS symptoms, as measured by:
  Subjective global assessment (SGA) of relief of IBS symptoms at 3, 6, and 12 months [24]
  A weekly response to the question “Have you had adequate relief of your IBS symptoms?”

3. IBS-associated somatic symptoms, as measured by the Patient Health Questionnaire-12 (PHQ-12) at 6 months [25, 26]
4. Anxiety and depression scores, as measured by the Hospital Anxiety and Depression Scale (HADS) at 3, 6, and 12 months [27]
5. Ability to work and participate in other activities, as measured by the Work and Social Adjustment Scale (WSAS) at 3, 6, and 12 months [28–30]
6. Acceptability of treatment, as measured by patient-reported choice to continue trial medication beyond 6 months
7. Self-reported adherence to treatment at 3 weeks and 3, 6, 9, and 12 months
8. Tolerability of treatment, as measured by self-reported adverse events (AEs) using the Antidepressant Side Effect Checklist (ASEC) at 3, 6, and 12 months [31]

#### Cost-effectiveness objectives

32To assess the effect of amitriptyline on:

1. Self-reported health care use, as measured by a health resource use questionnaire at 3, 6, and 12 months
2. Health-related quality of life, as measured by the EQ5D-3L at 3, 6, and 12 months [32, 33]
3. Cost-effectiveness, as measured via the incremental cost-effectiveness ratio and expressed in terms of incremental cost per quality-adjusted life year (QALY) at 6 and 12 months

#### Internal pilot objectives

To assess recruitment and 6-month follow-up rates against pre-defined progression criteria.

#### Nested qualitative study objectives

1. To understand patient and GP experiences of treatments and trial participation, and how these can inform our understanding of the quantitative results and future implementation in primary care
2. To identify factors that facilitate or impede prescribing and acceptability of, as well as adherence to, low-dose amitriptyline in this patient group
3. To identify patient and GP perspectives on the broader impact of the trial, including any unanticipated effects not captured by the quantitative measures
4. To explore psychosocial and contextual factors that might shape the wider use of amitriptyline for IBS

### Trial design

ATLANTIS is a randomised, multi-centre, parallel-group, two-arm, double-blind, placebo-controlled superiority trial of low-dose amitriptyline as a second-line treatment for people with IBS in primary care. Participants will be randomised 1:1 to receive amitriptyline or placebo for 6 months. At 6 months, patients will be offered the option of continuing their allocated trial medication for a further 6 months (see the “Intervention” section for protocol amendment V5.0). Participant-reported assessments are conducted at baseline, 3, 6, and 12 months post-randomisation. An internal pilot will assess pre-defined progression criteria for recruitment and follow-up rates. A nested, qualitative study will explore patient and GP experiences of treatments and trial participation.

## Methods

### Trial setting

Patients will be recruited from approximately 75 general practices, within urban and rural settings, with a range of socio-demographic and diversity characteristics, across three UK geographical regions, referred to as “hubs”: West Yorkshire, Wessex, and the West of England. Each practice is classed as a research site with a GP principal investigator (PI). Practices will be required to have obtained practice management approval and undertake a site initiation visit prior to recruiting to the trial. Patients will be directed to respond to the invitation to the main hub research team, comprising a “hub lead clinician” and research nurse or clinical study officer (CSO), responsible for coordinating patient screening and activity.

### Eligibility criteria

 Eligible patients must meet all inclusion criteria and none of the exclusion criteria listed in Table 1.

**Table 1:** Eligibility criteria

**Inclusion criteria**
1. Aged ≥ 18 years with a diagnosis of IBS (of any subtype of stool pattern [diarrhoea, constipation, mixed bowel habit, or unclassified]) in their primary care record, and fulfilling the Rome IV criteria for IBS 2. Ongoing symptoms, defined as an IBS symptom severity score (IBS-SSS) score of ≥ 75 at screening, despite being offered dietary advice and first-line therapies, as defined by the NICE guideline (antispasmodics [e.g. mebeverine], fibre supplements [e.g. fybogel], laxatives [e.g. bisacodyl], or anti-diarrhoeals [e.g. loperamide]), assessed at screening via patient self-report 3. A normal haemoglobin, total white cell count, and platelets within the last 6 months prior to screening 4. A normal C-reactive protein within the last 6 months prior to screening 5. Exclusion of coeliac disease, via anti-tissue transglutaminase antibodies, as per NICE guidance 6. As amitriptyline is harmful in overdose, patients must have no evidence of active suicidal ideation, as determined by three clinical screening questions, and no recent history of self-harm (within 12 months prior to screening). All patients will be asked (1) whether they have experienced any thoughts of harming themselves, or ending their life in the last 7–10 days; (2) whether they currently have any thoughts of harming themselves or ending their life; and (3) whether they have any active plans or ideas about harming themselves, or taking their life, in the near future. These questions are used in preference to a formal suicidal risk rating scale, as such scales perform poorly in clinical practice. Any positive response on any of the questions will trigger an urgent GP review 7. If female, patients must be post-menopausal, or surgically sterile, or using highly effective contraception (and must agree to continue for 7 days after the last dose of the investigational medicinal product [IMP]) 8. Able to complete questionnaires and trial assessments and to provide written informed consent
**Exclusion criteria**
1. Age > 60 years with no GP review in the 12 months prior to screening (a safety criterion due to the increased risk of gastrointestinal pathology > 60 years to ensure any changes in bowel habit are IBS-related and do not require further investigation) 2. Meeting locally adapted NICE 2-week referral criteria for suspected lower gastrointestinal cancer 3. A known documented diagnosis of inflammatory bowel disease or coeliac disease 4. A previous diagnosis of colorectal cancer 5. Individuals participating currently, or who have within the 3 months prior to screening been involved in, another clinical trial of an investigational medicinal product 6. Pregnancy, breastfeeding, or planning to become pregnant within the next 18 months 7. Current use of a TCA, or use of a TCA within the last 2 weeks prior to randomisation, for another indication 8. Allergy to TCAs or any other known contraindication to the use of TCAs. The latter includes taking monoamine oxidase inhibitors, or receiving them within the last 2 weeks; already receiving a TCA for the treatment of depression; previous myocardial infarction; recorded arrhythmias, particularly heart block of any degree, or prolonged Q-T interval on an ECG; mania; severe liver disease; porphyria; congestive heart failure or coronary artery insufficiency; or receiving concomitant drugs that prolong the QT interval (e.g. amiodarone. fluconazole, or terfenadine)

### Recruitment, randomisation, and blinding

#### Recruitment

General practices interested in participating will be identified via expression of interest with the support of the local Clinical Research Networks (CRNs). Participating practices will search their patient list for potentially eligible adult patients, using a SnoMed code list developed previously (Additional file 1: example electronic health record search). Potential participants will be invited to take part via mailed letter, opportunistic recruitment during GP consultations, and advertisements for self-referral placed in participating general practices, community pharmacies, and on practice/specialist IBS organisational websites. Interested patients will either return a reply slip or contact the recruiting hub via email or telephone, or be directed to the ATLANTIS website containing a self-screening questionnaire with contact details for the local recruitment hubs. Interested patients will be screened by the hub research nurse or CSO against initial eligibility criteria on the telephone. Those identified as potentially eligible will be invited to a clinic appointment to provide informed written consent with the hub research nurse, CSO, GP research nurse, or CRN nurse (trained in good clinical practice (GCP) and trial procedures); have screening bloods taken (including a full blood count and C-reactive protein (if not tested within the previous 6 months) and anti-tissue transglutaminase antibodies for coeliac screening (if not previously tested)); and be registered into the trial (Additional file 2: Patient information sheet and consent form). In view of the potential lethality of amitriptyline in overdose, three brief screening questions will be used to identify any potential participants with suicidal ideation, who will then be referred to their GP for further assessment.

The general practice PI will review the blood test results and patient screening to confirm trial eligibility, which is further checked by the hub lead clinician. Participants confirmed as eligible will then complete the baseline questionnaires and be randomised subsequently.

#### Randomisation

Randomisation will be performed by the hub research nurse or CSO via an automated system based at the University of Leeds Clinical Trials Research Unit (CTRU). Participants will be allocated to amitriptyline or placebo via minimisation incorporating a random element to ensure the treatment arms are well balanced for depression score (HADS-depression score ≥ 8), predominant stool pattern (diarrhoea, constipation, mixed bowel habit, or unclassified), and hub. Following randomisation, an automatically generated email will be sent to the central pharmacy with instructions to dispense specific trial medication kits, depending on the participant’s allocation, which will be posted directly to the participant.

#### Masking

Neither the participant nor those responsible for their care or evaluation (the primary care team and the research nurses or CSOs) will know the treatment allocation. This will be achieved by identical packaging and labelling of both amitriptyline and placebo, and the use of unique kit codes.

The ATLANTIS trial team and CTRU personnel involved in the day-to-day running of the trial will be masked to group allocation until the final database lock. A CTRU trial safety team will have access to treatment allocation, throughout the trial duration, for participant unmasking and preparation of unmasked safety reports to the Data Monitoring and Ethics Committee (DMEC).

Participants will be able to request for themselves and their GP to be told their treatment allocation shortly after their final follow-up at 12 months, to facilitate post-trial treatment decisions. Treatment allocation will be provided via email, supported by an evidence-based leaflet developed with input from patient and public involvement (PPI) representatives, only when CTRU have confirmation that all study assessments and contacts are complete, and all required data have been received. This will remain confidential (to the participant and their GP), as far as possible, to protect and maintain the overall masking for the trial research team. Should participants need support following provision of treatment allocation, they will be directed to the ATLANTIS qualitative researcher who is independent from the day-to-day running of the trial, recruitment, data collection, and treatment decisions.

Emergency unmasking (before 12 months) may be requested on the grounds of safety, when information about the participant’s trial treatment is critical for their ongoing clinical care, by the chief investigators (CIs), local PI, authorised delegate, or treating physician. Treatment allocation will be revealed by the CTRU using code-break envelopes for office hours requests, with a duplicate copy being held at the central pharmacy for out-of-hours requests.

### Intervention

Patients will be provided with written guidance on how to titrate their trial medication. They will be advised to commence at a dose of 10 mg (one tablet) daily with dose titration over 3 weeks, up to a maximum of 30 mg at night (three tablets), depending on side effects and response to treatment. After an initial 3-week titration, it is expected that the majority of participants will remain on a steady dose, but they can modify their dose throughout the study in response to their IBS symptoms and any side effects, reflecting how amitriptyline is used in usual care. All participants consent to 6 months of trial medication. They will be offered the option of continuing their allocated treatment for a further 6 months (although this will not be offered to some later recruits due to truncated follow-up for participants who were randomised during the last 6 months of the trial).

For safety purposes, due to the risk of harmful overdose, an initial 1-month supply of trial medication will be sent to participants by the central pharmacy following randomisation. Further trial medication will be dispensed at months 1, 3, 6, and 9 (as appropriate) and ordered by the hub research nurse or CSO using the CTRU web-based kit allocation system. The hub research nurse or CSO will contact participants by telephone at weeks 1 and 3, and months 3, 6, 9, and 12 to assess adherence to trial medication and concomitant medications and provide support as needed. All participants have the option of a GP consultation approximately 1 month after commencing trial medication to provide support if they have queries or concerns.

### Relevant concomitant care permitted or prohibited during the trial

Usual care for IBS will be provided by the participant’s GP, with the exception that amitriptyline or other TCAs cannot be commenced during the trial. The use of monoamine oxidase inhibitors and drugs that prolong the QT interval (e.g. amiodarone, terfenadine, or sotalol) are prohibited for the duration of the trial.

### Provisions for post-trial care

Following trial and outcome measure completion, participants will have the option to discuss amitriptyline prescription for IBS under the care of their GP if they wish.

### Trial assessments and data collection

**Fig. 1:**
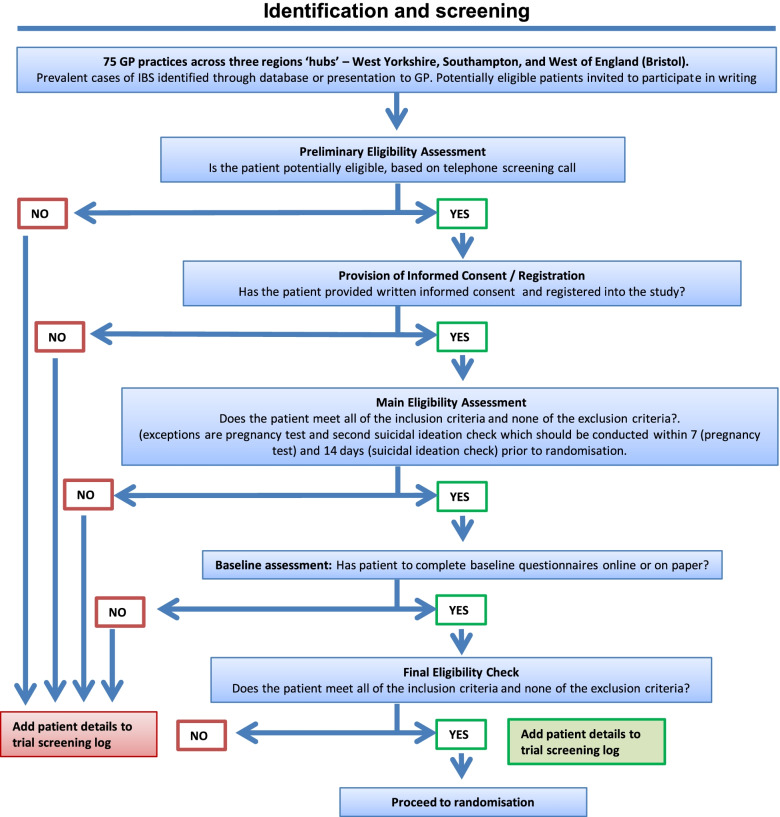
Trial flow chart — participant identification and screening

**Fig. 2:**
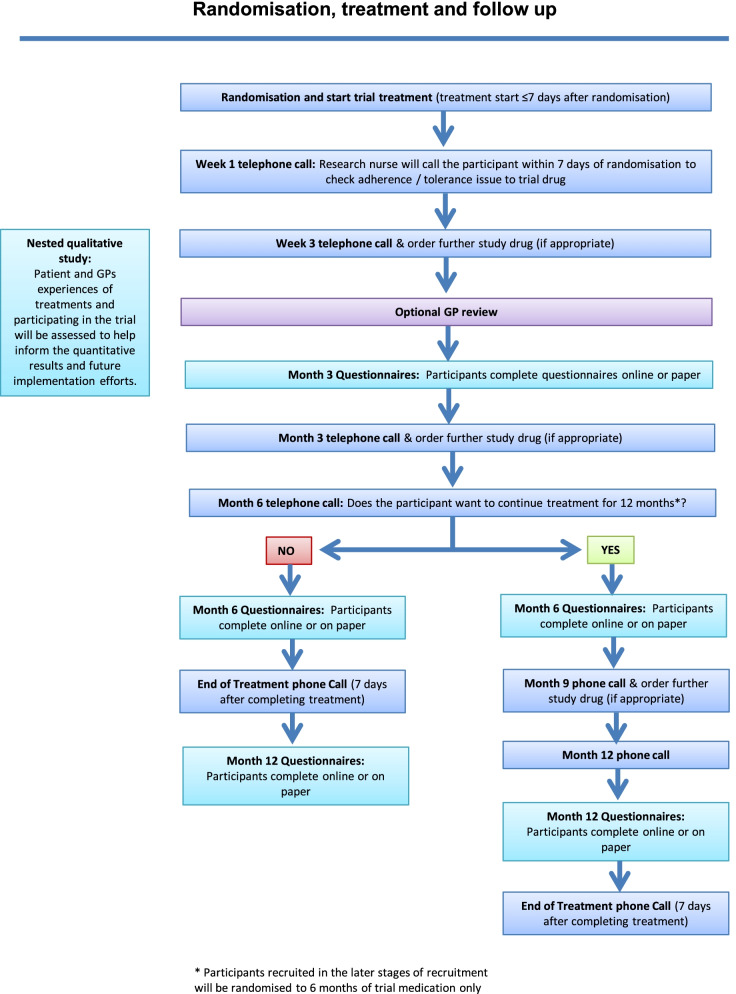
Trial flow chart — randomisation, treatment, and follow-up. For participants recruited following Protocol v5.0, follow-up concludes at 6 months

Required data, assessment tools, collection time points, and processes are summarised in Figs. 1 and 2. Additional file 3 provides further information on each of the outcome measures and methods of data collection. Data will be collected via paper or electronic case report forms and questionnaires or electronically via the CTRU automated registration/randomisation/kit system and the electronic software “REDCap”.

All participants will be asked to complete questionnaires at baseline and at 3, 6, and 12 months post-randomisation (unless recruited after protocol amendment v5.0) and to answer a weekly question “Have you had adequate relief of your IBS symptoms?” for 6 months. Participants will be sent text message and email reminders after 1 week to prompt completion. The hub trial team will telephone the patient to collect outstanding data for all outcomes for non-responders.

### Safety

Participants will self-report adverse events (AEs) to the hub research nurse/CSO via the ASEC at weeks 1 and 3 and months 3, 6, 9, and 12. All serious adverse events (SAEs) and suspected unexpected serious adverse reactions (SUSARs) will be collected throughout the study, from the time of randomisation until 7 days following discontinuation of the study drug. All reportable AEs, SAEs, and SUSARs (the latter considered highly unlikely in this trial) will be reported to the independent DMEC and relevant regulatory bodies within the required time frames, and appropriate action will be taken.

### Nested qualitative study

#### Design

Maximum variation samples of trial participants and GPs from participating practices will be invited to take part in semi-structured audio-recorded telephone interviews. Patients will be interviewed twice, at approximately 6 and 12 months post-randomisation. GPs will be interviewed once, after completing initial patient recruitment at their practice. Interviews will be conducted after obtaining informed consent and will be anonymised on verbatim transcription. Topic guides comprising open-ended questions and follow-up prompts will be used flexibly to explore individual experiences and perspectives in-depth and to allow novel and unanticipated insights to emerge. Field notes will capture the interviewer’s impressions and reflections. Data collection and analysis will proceed iteratively; the final sample sizes will depend on saturation and when we achieve a rigorous, credible analysis in relation to the qualitative aims.

#### General practitioner interviews

We will aim to interview a diverse range of approximately 30 GPs (full and part-time, sex, rural/urban practice, practice deprivation index, and years as a GP). Interviews will explore GPs’ experiences of the use of amitriptyline prior to and within the trial (in the broader contexts of management of IBS symptoms and other conditions), and potential barriers and facilitators to widespread post-trial implementation in primary care. The topic guide will be informed by relevant literature, key domains from normalisation process theory (NPT) [34], and discussions with the PPI group. NPT provides a framework that specifies the factors and processes likely to hinder or enable widespread implementation of new practices.

#### Participant interviews

We will aim to interview a diverse range of approximately 20 participants from each arm of the trial (to encompass participants from each hub, a mix of sex and ages, a range of baseline symptom severity scores according to the IBS-SSS, and to include those who have decided to continue or stop treatment at 6 months). The interviewer will remain blinded to treatment allocation at the time of the interviews.

The interviews will explore participants’ experiences of recruitment, randomisation, and the trial in general and experiences of initiating and (dis)continuing trial medication and its perceived impacts and changes over time. The 6- and 12-month topic guides will be informed by relevant literature, the common-sense model of illness perception [35], and discussions with our PPI group. The common-sense model provides a framework for understanding how participants experience treatments and make treatment decisions within the context of chronic illness; this has proved relevant in previous qualitative work on IBS [36].

### Sample size

A sample size of 414 evaluable patients will provide 90% power to detect the minimum clinically important between-group difference of 35 points on the IBS-SSS [37], between amitriptyline and placebo at 6 months, assuming a standard deviation of 110 points [38, 39] and 5% significance level. This equates to a small to moderate effect size of 0.32. This 35-point between-group difference was the agreed minimum clinically important between-group difference for the IBS-SSS in another large UK treatment trial in IBS in primary care, the ACTIB trial [37, 40]. The sample size also gives at least 85% power to detect a 15% absolute difference in the key secondary outcome (SGA of relief of IBS symptoms) [24]. Uptake of drugs for IBS that provide a lower therapeutic gain over placebo is less likely [41, 42]. We aim to recruit 518 participants to allow for a 20% loss to follow-up.

### Internal pilot

An internal pilot, commencing in the first month of trial recruitment, across all three hubs, will assess recruitment at 6 months (reviewing rates over the previous 3 months) according to the criteria in Table 2.

**Table 2:** Internal progression criteria

Criteria	Green/continue	Amber/review	Red/stop
Monthly recruitment rate (averaged over months 4–6 of internal pilot)	≥ 80% of the target of 28 pts/month: ≥ 22.4	50–80% of 28 pts/month: 14–22.4 pts/month	< 50% of target of 28 pts/month: < 14 pts /month
Follow-up for 6-month primary outcome	≥ 80%	60–80%	< 60%
Outcome of progression review	The study will continue and outcome data from participants in the internal pilot will be included in the main study analysis	A rescue plan will be developed and approved by the TSC before submission to the funder	The TSC will consider not progressing the internal pilot to the definitive study

### Data analysis

#### General considerations

A detailed statistical analysis plan will be written and signed off before the analysis is undertaken. All analyses will be conducted on the intention-to-treat population, defined as all participants randomised, regardless of adherence to the intervention. An overall two-sided 5% significance level will be used for all endpoint comparisons. Outcome data will be analysed once only after data lock, at the final analysis, and no interim analyses are planned.

#### Primary analysis

A linear regression model, adjusted for minimisation variables and IBS-SSS score at baseline, will be used to test for differences between the treatment groups on the IBS-SSS at 6 months. The IBS-SSS is a validated, patient-reported, 5-item questionnaire widely used in trials of medical therapies in IBS [23]. Missing data will be imputed via multiple imputation, where appropriate. Sensitivity analyses on a per-protocol population and on participants with complete data will test robustness of results. Results will be expressed as point estimates, together with 95% confidence intervals and *p*-values.

#### Secondary analyses

Continuous endpoints at 6 months (HADS, WSAS, and PHQ-12 scores) will be analysed in the same manner as the primary endpoint adjusted for the respective baseline score. Secondary binary endpoints (SGA of relief of IBS symptoms, acceptability of treatment, continuation of trial medication, and adherence) at 6 months will be analysed similarly in logistic regression models.

Additional exploratory analyses of all endpoints at 3 and 12 months will be carried out, as well as repeated measures models incorporating all time points. IBS symptoms reported weekly will be analysed using a repeated measures model. Exploratory moderator analyses will be conducted to investigate if the 6-month treatment effect on the IBS-SSS varies by IBS subtype or mood by including an interaction between the treatment arm and each potential moderator in the primary analysis model.

#### Safety analyses

All participants receiving at least one dose of trial medication will be included in the safety analysis. Descriptive statistics of self-reported AEs on the ASEC will be presented by arm. The number of participants reporting a SAE and details of all SAEs will be reported for each treatment group. The number of participants withdrawing from trial treatment will be summarised by treatment arm, along with reasons for withdrawal.

#### Cost-effectiveness analyses

A within-study cost-effectiveness analysis will be conducted adopting the perspective of the NHS and Personal Social Services and a societal perspective. The time horizon will be 6 months; hence, costs and outcomes will not be discounted. The primary outcome will be cost per QALY. We will assess uncertainty using a within-trial probabilistic sensitivity analysis undertaken using Monte Carlo simulation, with results presented as incremental cost-effectiveness ratios and cost-effectiveness acceptability curves, assuming a willingness to pay (lambda) of £20,000 per QALY. Sensitivity analyses will include a 12-month horizon, as well as a scenario as close as possible to a real NHS context, where the treatment is prescribed by the GP with repeated prescriptions, tests, and required appointments. Health economic analyses will be subject to further funding (protocol v5.0).

#### Qualitative analysis

Interviews will be transcribed verbatim (by professional transcribers and/or the research team) and transcripts will be anonymised. Transcripts will be imported into NVivo to facilitate data management and record-keeping. We will employ established techniques to enhance the quality and credibility of our analysis, including maintaining an audit trail to ensure transparency, involving multiple individuals — including PPI representatives — to ensure diverse perspectives are brought to bear on the data and avoid idiosyncratic interpretations, and deliberately seeking out anomalous or “deviant” cases and using them to identify important, but rare, views and the limits of the analysis. Our themes will be based on importance and relevance, not prevalence, consistent with best practice in qualitative research involving in-depth analysis of small, diverse samples.

Thematic analysis [43] will proceed separately for GP and patient interviews and will be augmented with coding techniques from grounded theory (e.g. open coding, line-by-line coding, constant comparison) [44]. Analysis will be primarily inductive, but we will draw on NPT (GP interviews) and the common-sense model of illness perception (patient interviews) to assist in interpreting findings related to wider implementation of amitriptyline for IBS in primary care and participants’ experiences of IBS and its treatment. For patient interviews, after having identified initial themes, and final trial unblinding, we will construct cross-tabulations (“matrices”) of themes by trial arm to help us relate the qualitative findings to the quantitative results.

### Patient and public involvement

The trial team includes a co-applicant and provides patient and public involvement (PPI) representation on the Trial Management Group (TMG) who leads a local IBS support group and a PPI officer who leads on the development and implementation of PPI throughout this study. In addition, there is PPI representation on the Trial Steering Committee (TSC), to ensure that a patient perspective is included in all decisions related to the trial. Patients will advise on design considerations, patient leaflets, invitation letters, recruitment strategies, the trial protocol, and dissemination activities. PPI will also be key in guiding the development of GP and patient interview topic guides. During recruitment, PPI perspective will be sought on emerging issues. We will offer free places on the CTRU “Introduction to Clinical Trials” training and financial reimbursement will be offered in line with INVOLVE guidance.

### Research governance

Trial supervision will be established according to the principles of GCP and in-line with the UK Policy Framework for Health and Social Care Research. This will include the establishment of a core project team, TMG, TSC, and DMEC.

The trial is managed on a day-to-day basis by the CIs and a core team at the Leeds CTRU. The TMG comprises the CIs, all co-applicants, hub study nurses or CSOs, the CTRU team, and the PPI representative, who meet monthly to review progress.

Independent oversight is provided by the TSC and DMEC. The TSC meets at least annually and is responsible for providing overall supervision for the trial in accordance with pre-agreed terms of reference, particularly trial progress, including completion of the internal pilot according to pre-defined success criteria and adherence to the protocol. The DMEC receives 6-monthly unblinded safety summaries and meets the trial team annually to monitor study conduct, participant safety, and unblinded data, in accordance with pre-agreed terms of reference. The DMEC may recommend discontinuation of the trial if significant ethical or safety concerns arise prior to trial completion.

### Monitoring and audit

As this trial is using a drug (amitriptyline) with a well-established safety profile and at a low dose, all monitoring for this trial will be done centrally at the CTRU throughout the conduct of the trial, with site monitoring only occurring for cause-triggered visits only.

### Confidentiality

All participant information collected during the trial will be kept confidential, in compliance with the 2018 Data Protection Act. Participant information will be held on a secure password-protected database. All trial documentation will be held in secure offices. At the end of the trial, all data held by the CTRU, and all trial data will be securely archived at the University of Leeds in line with the trial sponsor’s procedures. Access to trial data will be subject to agreement with the Leeds CTRU and the ATLANTIS CIs, through a legally binding contract with the University of Leeds.

### Dissemination policy

Results of the trial will be disseminated via traditional academic routes and by social media and patient groups. Authorship guidelines will be developed. Results will be presented at national and international primary care and gastroenterology meetings and, therefore, will be cascaded to GPs and gastroenterologists. As well as being published in the publicly available HTA journal, we plan to publish our research in high-impact, high-quality, multi-disciplinary journals. With the help of our PPI group, we will produce plain English summaries and podcasts of findings available online for relevant patient groups and trial participants.

## Discussion

The ATLANTIS trial will be the first RCT of amitriptyline in IBS to be conducted in primary care, and the largest trial of a TCA for IBS to date. This trial will have a real impact on clinical practice, as it will identify whether amitriptyline is effective for the management of IBS in this setting, improving the management of IBS in primary care. If effective, amitriptyline may improve symptoms, quality of life for patients and, ultimately, lower overall costs to the health service for the management of IBS. If amitriptyline is cost-effective as a second-line management strategy for IBS, then this will have important implications for the health service.

The challenges we expect this trial to present mainly relate to patient recruitment and retention. Early discussions with PPI identified the possible reluctance some patients may have to take amitriptyline. Amitriptyline is most well recognised as an antidepressant drug. However, these effects are present at doses much higher than the maximum dose of 30 mg being used in this trial. The usual doses to treat depression are 75 to 150 mg/day and up to 225 mg to 300 mg/day for severe resistant depression. Our PPI activity made recommendations to ensure understanding of the rationale for its use in IBS and was involved in developing clear written guidance on how to dose titrate, as well as education about potential side effects, which will hopefully tackle potential misconceptions, increase adherence to trial medication, and minimise drop-outs.

## Trial status

ATLANTIS recruited its first participants in December 2019. However, the COVID-19 pandemic resulted in the trial pausing recruitment in March 2020 for 4 months and led to subsequent reduced rates of new practice and participant recruitment. In light of this, internal pilot objectives were difficult to evaluate in the original timeframe and a costed trial extension is required to complete recruitment and follow-up of the trial. An extension to permit recruitment to the end of March 2022 has been granted by the funder. Several substantial amendments have been made to the trial protocol, including an approved amendment due to COVID-19 impact on trial recruitment timelines to reduce the duration of trial medication and follow-up from 12 to 6 months and to remove the cost-effectiveness analysis (now dependant on further additional funding) to minimise additional funding required to complete the trial. Site and hub PIs, hub researchers, and participants (where relevant) have been, and will be, informed of all protocol amendments following ethical and regulatory approvals. Trial registries and journals will be informed as appropriate.

## Supplementary Information

**Additional file 1.** Electronic health record search.

**Additional file 2.** Participant information sheet and informed consent form.

**Additional file 3.** Outcome measures and methods of data collection.

**Additional file 4.** REC Approval Letter.

## Data Availability

The University of Leeds will govern access to the data; data can be shared with collaborators for the purpose of delivering the project. Access to data for other purposes will require approval by the TSC during the project, or by the co-CIs, after the project is completed.

## Abbreviations

AE: Adverse event
ASEC: Antidepressant Side-Effect Checklist
CI: Chief investigator
CRN: Clinical Research Network
CTRU: Clinical Trials Research Unit
DMEC: Data Monitoring and Ethics Committee
GP: General practitioner
HADS: Hospital Anxiety and Depression Scale
IBS: Irritable bowel syndrome
IBS-SSS: IBS Severity Scoring System
NICE: National Institute for Health and Care Excellence
PHQ-12: Patient Health Questionnaire-12
PI: Principal investigator
PPI: Patient and public involvement
QALY: Quality-adjusted life year
RCT: Randomised controlled trial
SAE: Serious adverse event
SGA: Subjective global assessment
SUSAR: Suspected unexpected serious adverse reactions
TCA: Tricyclic antidepressant
TMG: Trial Management Group
TSC: Trial Steering Committee
WSAS: Work and Social Adjustment Scale
