# Supplementary material for: Amitriptyline at low-dose and titrated for irritable bowel syndrome as second-line treatment (The ATLANTIS trial): protocol for a randomised double-blind placebo-controlled trial in primary care
Source: Trials. 2022 Jul 8;23:552. doi: 10.1186/s13063-022-06492-6 (PMC9264306; doi:10.1186/s13063-022-06492-6)
Supplement: Supplementary file 1 — Additional file 1. Electronic health record search. [file 13063_2022_6492_MOESM1_ESM.pdf]

Mandatory In
Optional In
Not In

ATLANTIS Final patient list
ATLANTIS

- NOT IN → **ATLANTIS Exclusions**
  ATLANTIS
  - IN → **TCAs current**
    ATLANTIS
    Has medication in the 'Tricyclic and related antidepressants' Action Group
    - Include all drug types
    - Start date after 92 days ago
  - OR IN → **pregnancy**
    ATLANTIS
    Has a code of Pregnant (77386006) or one of its children
    - Using children from the SNOMED hierarchy, including inactive children.
    - Event was entered after 365 days ago
  - OR IN → **palliative care dementia care home**
    ATLANTIS
    Has a Read code in...Codes and Children:
    Lives in staffed home (Ua0Lj)
    Dementia (X002w)
    Palliative care (XaEJE)
  - OR IN → **MI ihd ccf porhyria mania**
    ATLANTIS
    Has a Read code in...Codes and Children:
    Cardiac arrhythmia (698247007)
    Heart failure (84114007)
    Ischaemic heart disease (414545008)
    Long QT syndrome (9651007)
    Mania (231494001)
    Myocardial infarction (22298006)
    Porphyria (418470004)
    Using children from the SNOMED hierarchy, including inactive children.
  - OR IN → **medication - MOA**
    ATLANTIS
    Has medication in the 'Monoamine-oxidase inhibitors (MAOIs)' Action Group
    - Include all drug types
  - OR IN → **DSH**
    ATLANTIS
    Has a Read code in...Codes and Children:
    Suicidal thoughts (1BD1.)
    Suicide attempt (Ua18F)
    Suicide risk (Ua1WE)
    Thoughts of deliberate self harm (Xalux)
    - Event was entered after 365 days ago
  - OR IN → **colorectal cancer**
    ATLANTIS
    Has a code of Malignant tumour of large intestine (363510005) or one of its children
    - Using children from the SNOMED hierarchy, including inactive children.
  - OR IN → **coeliac disease & IBD**
    ATLANTIS
    Has a Read code in...Codes and Children:
    Ulcerative colitis (XE0ag)
    Coeliac disease (XE0bK)
    Crohn's disease (XE2QL)
  - OR IN → **allergy TCA**
    ATLANTIS
    Has a sensitivity in the 'Tricyclic and related antidepressants' action group
- AND IN → **IBS aged >18 in past 3 years**
  ATLANTIS
  Has a Read code in...Exact Codes:
  Irritable bowel syndrome with diarrhoea (J5210)
  Irritable bowel syndrome (XE0as)
  Management of irritable bowel syndrome

(XaYnp)
Irritable bowel syndrome characterised by constipation (XabiP)
IBS characterised by alternating bowel habit (XabiQ)
Current age > 18 years
