# Supplementary material for: Amitriptyline at low-dose and titrated for irritable bowel syndrome as second-line treatment (The ATLANTIS trial): protocol for a randomised double-blind placebo-controlled trial in primary care
Source: Trials. 2022 Jul 8;23:552. doi: 10.1186/s13063-022-06492-6 (PMC9264306; doi:10.1186/s13063-022-06492-6)
Supplement: Supplementary file 2 — Additional file 2. Participant information sheet and informed consent form. [file 13063_2022_6492_MOESM2_ESM.pdf]

Amitriptyline at Low-dose and Titrated for Irritable Bowel Syndrome as Second-line Treatment: The ATLANTIS study

PARTICIPANT INFORMATION SHEET AND INFORMED CONSENT FORM

You have been invited to take part in a research study called “ATLANTIS”. Before you decide if you want to take part, we would like to give you some more information about the study.

Please read this information carefully, and feel free to discuss it with others if you like. Please ask us if anything is unclear, or if you would like more information.

If you are interested in taking part, please return the reply slip in the prepaid envelope enclosed with this letter. Alternatively, you can email the research team using the contact details provided in this document. A researcher will then contact you to talk about the study and answer any questions you may have.

This leaflet is split into 2 parts:

- **Part 1** tells you why we are doing the study and what will happen to you if you decide to take part.
- **Part 2** gives you more detailed information about how the study will be run.

How to contact us

If you have any questions about this study, please talk to the researcher at

<<Enter recruitment hub researcher name >>

<< Contact details for recruitment hub>>

Thank you for reading this information sheet.

FUNDED BY

NIHR | National Institute for Health Research

Part 1

Why are we doing this study?

Irritable bowel syndrome (IBS) is usually a long- term problem and can have a significant impact on people's lives. Unfortunately, we know that current IBS medications do not help everyone. Amitriptyline is a drug that can be used to treat IBS. It is recommended in UK guidelines for people who continue to have troublesome symptoms, because small studies of amitriptyline have shown promising results. This study aims to see whether the drug helps patients with IBS who are looked after by their GP. Nobody has done a large study to explore this before, and we hope the results will lead to more effective treatment for IBS. The results from this study are important, as they will help doctors decide whether they should be using amitriptyline routinely as a treatment for IBS.

What is amitriptyline?

Amitriptyline is a frequently used drug, which has been in use for more than 50 years. We believe it helps with IBS at a low dose because it relieves pain and changes bowel activity. Amitriptyline is sometimes used to treat depression. However, when being used for depression, it is given in much higher doses. This study will only be using small doses of amitriptyline, and is being used because of its impact on pain, rather than mood. As with any medication, there are some potential side effects. These are listed in the second part of this leaflet.

What is a placebo?

Half the people who take part in the study will be asked to take amitriptyline. The other half will be asked to take a dummy tablet known as "placebo." A computer will randomly decide which you are given. There is more information about this process in Part 2 of this leaflet.

Why are we using a placebo?

We are using a placebo in this study because we need to find out if amitriptyline works for people with IBS. IBS symptoms vary over time and it is possible that improvements may be due to factors other than the treatment. Comparing those on amitriptyline with those on placebo allows us to work out how much of the improvement seen is because of amitriptyline.

Will I be told if I am having amitriptyline or placebo?

Yes, you will be able to find out after 6 months if you have been taking amitriptyline or placebo. We are not able to tell you before 6 months as this may affect the results of the study (an important part of this study is how you feel and this may be affected by knowing which treatment you have received). However, if there is an urgent reason your GP needs to know what treatment you have received, they will be told.

Why have I been invited to take part in this study?

You are being invited to take part because your GP records show that you have IBS. Your GP practice has agreed to take part in this study. We

are hoping to recruit more than 500 people with IBS from approximately 75 GP practices across West Yorkshire, Southampton, and the West of England.

Do I have to take part?

No, your participation is entirely voluntary and it is up to you to decide whether or not to take part.

If you decide to take part you will be given this information document to keep. You will be asked to sign a consent form, but you are still free to withdraw at any time and without giving a reason. If you decide not to take part, your GP will be happy to talk through alternative treatment options for your IBS. Your treatment and care will not be affected in any way.

If I want to, will I definitely be able to take part?

Unfortunately, no. Although your GP thinks you might be suitable to take part, we may still need to carry out a blood test and ask you some questions to make sure you are suitable. These are known as "eligibility screening tests" and are described below. If the eligibility screening tests show that it is not appropriate for you to take part in the study your GP will discuss alternative treatment options with you.

What will happen to me if I take part?

You will get a phone call from a researcher to check that you are suitable to take part in the study

If you are suitable to take part you may be asked to come to your GP practice to have a blood test

15 mins

You will be asked to complete an online or postal questionnaire about your IBS and how it impacts you

1 hour

You will be randomly allocated to amitriptyline or placebo

You will be asked to take the study drug for 6 months

You will receive a telephone call from the researcher after 1 week, 3 weeks, 3 months, and 6 months

You will be asked to complete an online or postal questionnaire in month 3 and month 6

You will answer a question once a week to let us know if the study drug is helping relieve your IBS symptoms

This completes Part 1 of the Information Sheet. If you are considering taking part, please read the extra information in Part 2 before making your decision.

Part 2

What do I have to do?

The diagram in Part 1 gives an overview of what you will be asked to do if you take part in the study. This section gives you a detailed breakdown of each part of the study.

Eligibility tests

- If you decide you would like to take part we would ask you to return the reply slip at the bottom of the invitation letter to the research team. Alternatively you can email the research team using the contact details provided.
- You will then have an initial telephone call with a researcher to find out more about you and your IBS to see if it is appropriate for you to take part in the study. The researcher may then need to check specific information about your medical history with your GP to make sure you are suitable to take part You may not be suitable to take part, for instance, if you have coeliac disease or inflammatory bowel disease, or if you have another condition which means you should not take amitriptyline (see page 8 of this information sheet for more detail)

- If you are eligible to take part you may be asked to come in to your GP practice and have some blood samples taken, to make sure you are not anaemic and that there are no signs that your bowel symptoms are due to other illnesses. The blood test is a straightforward, safe procedure but may cause some minor discomfort and you may notice some slight bruising which should go away in a couple of days. The blood samples will not be retained after testing is complete - they will be destroyed by the NHS laboratory conducting the testing according to their standard procedures.
- If you are a woman, able to have children, and are unable to confirm you are not pregnant, you will be provided with a pregnancy test to use at home before going into the study. This is to ensure it is safe for you to receive amitriptyline in the study.

Questionnaire

- You will then be asked to complete an online questionnaire or a paper questionnaire, which will ask you information about yourself, any past and current medications, how you are feeling, details about your IBS symptoms and how much of an impact it has on your daily life. The Clinical Trials Research Unit [CTRU] at the University of Leeds will email you a link to the online questionnaire or send the link to your mobile phone. Alternatively, a paper questionnaire can be posted to you.

Randomisation

- If you are eligible, you will be randomly chosen to receive either amitriptyline or placebo. Your supply of amitriptyline or placebo will be delivered to your home address by Royal Mail First Class recorded delivery. This will always be in plain packaging. You will initially receive a supply for 1 month, then a supply for 2 months and a final supply for 3 months.

Drug treatment

- You will start by taking 10 mg (1 tablet once a day at night) for 1 week. After the first week you will have the opportunity to discuss a dose change with the researcher and then you can decide whether you would like to stay on the 10 mg dose or increase your dose to 20 mg (2 tablets once a day at night). A final increase to 30 mg (3 tablets once a day at night) can be made during the third week.
- You will also be given a dietary advice sheet, which will advise of any foods you should avoid and how to manage your IBS symptoms.

Phone calls

- You will have a telephone call with a researcher in week 1, week 3, month 3 and month 6 (of taking the study drug) to give you advice about your study medication, and check if there are any problems, as well as to answer any questions you may have.

Questionnaires

- You will complete an online or postal questionnaire three times (before entering the study, then at 3 months, and 6 months after starting the study drug). To help you remember to complete the questionnaires, the research team at CTRU will send you reminders by email and text messages to your mobile phone. The research team in your local area may also attempt to contact you. If you do not have an email or mobile phone, the researcher may phone you.
- You will answer a question once a week to let us know if the study drug is helping relieve your IBS symptoms. This can be answered online or can be recorded in a paper diary, which will be collected at the end of the study. To help you remember to answer the question CTRU will send you text reminders to your mobile phone.

End of treatment

- You will have a telephone call with the researcher when you finish taking the study drug to check if there are any problems and to answer any questions you may have.

How long does the treatment go on?

You will be asked to take the study drug for 6 months. If you change your GP practice during the course of the trial, your new GP may not be able to prescribe the study medication.

Additional research

We would like to invite approximately 40 people to take part in a telephone interview to talk more about their experience in this study. If you are invited, you may be asked to take part in an interview, which will last approximately 1 hour. You will be sent more information about this if you are selected to take part.

You can choose to end the interview at any time and you do not have to answer any questions you do not want to.

If you would be happy to be approached for a telephone interview, there is an optional section on the consent form for you to complete. Even if you do not wish to be approached for these interviews, you can still take part in the ATLANTIS study.

Future research

The research data collected about you in the Atlantis study may be shared with other research teams to answer new research questions in the future. They will not be able to identify you from this data.

We would also like your permission to obtain information held within your electronic health records, including any hospital attendances and admissions and your health conditions from your GP and hospital records. We may use this for future research linked to the Atlantis study, or for other research projects. We would only do this once any future projects had received ethical approval subject to ethical approval. To obtain your health record data, we would need to send a limited amount of your identifiable data (for example, initials, data of birth, and NHS number) to the relevant data provider to obtain the correct information from these records. Other research teams may be involved in this future research but they will not be able to identify you from the information provided to them.

What are the possible side effects of the study medication?

Amitriptyline has been used widely for over 50 years and we are using it in a low dose in this study. However all medicines may have side effects. Common side effects include:

- constipation
- dizziness
- dry mouth
- feeling sleepy, tired or weak. This is the reason we ask you to take the study drug at night.
- difficulty peeing
- headache

If you are a woman who may become pregnant, you will be asked to confirm that you are not pregnant before taking part in the study, and may be asked to take a pregnancy test. You must also agree to use a reliable form of effective contraception during this time. If you do become pregnant during the study, or you find out that you are pregnant within three months of finishing treatment, then you must tell the study research team at once. Your GP will advise you on the potential risks to your unborn child and the options available to you.

What are other possible disadvantages of taking part?

Taking part in the study will involve time commitments for seeing and talking to the researcher, and completing questionnaires.

The study drug might harm an unborn baby; therefore you should not take part in this study if you are pregnant. You should not become pregnant during the study treatment period or for a safety period of at least 7 days after taking your last study treatment tablet.

What are the possible benefits of taking part?

We cannot promise the study will help you, but you may have improvements in your IBS symptoms, and the new knowledge we gain may help others with IBS. If the study shows amitriptyline to be effective it could become widely available from your GP in the near future, and help many patients like yourself. Taking part in this study will also give you the opportunity to receive extra health checks, via the blood tests, and to discuss your IBS symptoms with healthcare professionals.

Warnings and precautions to using the study medication

Amitriptyline should not be taken by patients who have:

- recently had a heart attack (myocardial infarction)
- heart problems such as arrhythmias (disturbances in heart rhythm which are seen on an electrocardiogram (ECG)), heart block, or coronary artery disease
- severe liver disease
- acute porphyria
- during the manic phase of bipolar disorder
- or if taking certain medications, including monoamine oxidase inhibitors (MAOIs).

The researcher will discuss this with you during your initial phone call about the study.

Please tell the researcher if you have, or have had in the past, any medical problems, so that they can check with your GP if you are suitable to take amitriptyline. In particular you should mention any of the conditions listed above, as well as the following below:

Cardiovascular disease; diabetes; epilepsy; history of psychosis or bipolar disorder; hyperthyroidism; increased intra-ocular pressure; phaeochromocytoma; prostatic hypertrophy (prostate gland enlargement); susceptibility to angle-closure glaucoma; difficulty passing urine;

What if relevant new information becomes available?

Sometimes during the course of a study, new information becomes available. If this happens we will tell you about it, and you will be able to decide whether you want to continue in the study. If you decide not to continue, your GP will continue your care. If you decide to continue you may be asked to sign an updated consent form. Occasionally, on receiving new information, your GP may consider it to be in your best interest to withdraw you from further study treatment.

Who has organised, reviewed, and funded the research and who will be supervising it?

The Chief Investigator is Professor Alexander Ford, based at the University of Leeds, who is supported by the Co-Chief Investigator Associate Professor Hazel Everitt based at the University of Southampton. The study is being sponsored by the University of Leeds, and is being organised

on their behalf by the University of Leeds CTRU. The University of Southampton and the University of Bristol, along with the University of Leeds, will manage and run the study in their local area (sometimes referred to as a 'hub'). The National Institute of Health Research fund the research. All research is looked at by an independent committee of people called a Research Ethics Committee to protect your interests. The study has been reviewed and approved by Yorkshire & The Humber – Sheffield Research Ethics Committee Ref: *19/YH/0150*.

What if there is a problem?

Emergencies:

In the unlikely event that you experience any problems during the study, we will have an emergency number, which would allow a doctor to find out if you were on amitriptyline or the dummy tablets, in case this might affect your treatment. You will also be given an identity card to carry with you, which details relevant information about the study, and contact numbers in case of an emergency.

Complaints:

If you have a concern about any aspect of this study, you should ask to speak with your researcher who will do their best to answer your question. If you remain unhappy and wish to complain formally, you can do this through the NHS Complaints Procedure. Details can be obtained from your GP practice or you can contact PALS (Patient Advice and Liaison Service) telephone << LOCAL PALS CONTACT >>

Harm:

Every care will be taken in the course of this clinical study. However, in the unlikely event that you are injured as a result of the managing organisation (University of Leeds), compensation may be available and you may have to pay your related legal costs. Your GP practice where you receive your treatment has a duty of care to you, whether or not you agree to participate in the study, and the University of Leeds accepts no liability for negligence on the part of your GP's practice. If you wish to complain about any aspect of the way you have been treated please contact your GP in the first instance.

Any claims will be subject to UK law and must be brought in the UK.

If you have private medical insurance, you should tell your insurer that you are taking part in research. They will let you know if it affects your policy.

What information will you collect about me?

As part of ATLANTIS we hope to:

1. Ask you to complete some questionnaires about you
2. Collect information about you from electronic health records, for example previous blood test results.
3. If you agree to take part, we may also ask you some questions about your experience.

Will my taking part in this study be kept confidential?

Yes. Under UK Data Protection laws the University of Leeds will act as the data controller

(legally responsible for the data security). This means they are responsible for looking after your information and using it properly.

The research teams at the University of Leeds, the University of Bristol, and the University of Southampton will collect information from you and handle this in accordance, with the 2018 Data Protection Act.

This information will be sent on paper forms to the CTRU at the University of Leeds (usually using standard Royal Mail but in some cases by secure email), or will be completed by you online and stored at the CTRU.

Where possible, information collected about you for the purposes of this research study (research data) will have your name and address removed, and a unique code, along with your initials and date of birth, will be used instead.

Your rights to access, change, or move your information are limited, as we need to manage your information in specific ways in order for the research to be reliable and accurate. You can find out more about how the University of Leeds uses personal data here: https://dataprotection.leeds.ac.uk/wp-content/uploads/sites/48/2019/02/Research-Privacy-Notice.pdf

Concerns and contact details

If you have any concerns about the way your personal data is being processed or have a query about the information in this leaflet, please contact the University of Leeds Data Protection Officer using any of the following details:

- Email: DPO@leeds.ac.uk;
- General postal address: University of Leeds, Leeds LS2 9JT, UK;
- Postal address for data protection issues: University of Leeds, Room 11.72 EC Stoner Building, Leeds, LS2 9JT;
- Telephone number: +44 (0)113 243 1751.

Our data controller registration number provided by the Information Commissioner's Office is Z553814X.

If you are not satisfied with our response, or believe we are processing your personal data in a way that is not lawful, you can complain to the Information Commissioner's Office (https://ico.org.uk/).

If you withdraw from the study, we will keep the information about you that we have already obtained, and this will still be used in analysing the results of the study. To safeguard your rights, we will use the minimum personally-identifiable information possible.

Your personal data (your name, NHS number, address, telephone number(s) and email address (if you have one) will be provided to the hub research team and CTRU. They will use this information to contact you about the research study (for example, to remind you to complete questionnaires), make sure relevant information about the study is recorded for your care, and to oversee the quality of the study

Your data will be entered onto secure databases held at the CTRU. Only data collected to answer the research question, to ensure the study is being run correctly or for safety reasons will be entered.

At the start of the study, a prescription will be posted from your GP to Leeds Teaching Hospitals Trust (LTHT) pharmacy to allow them to dispense and post the study medication to you. This prescription will include your name, date of birth, NHS number, home address, and study number. A copy may also be sent by fax or email. During the study, if your address changes this information will be collected by the researcher and sent to LTHT pharmacy to ensure study medication is sent to the correct address.

At the end of the study, your personal data and research data will be securely stored at the University of Leeds. Data pertinent to your medical care will also be stored in your medical records at your GP practice, even if you stop taking part in the study. Only those who need to will have access to the data.

After the end of the study your data will be stored securely for a minimum of 25 years. After this time your data will be disposed of securely.

Data access

The data collected for the study will be looked at and stored by authorised persons from the research teams at the University of Leeds, the University of Bristol, and the University of Southampton. They may also be looked at by authorised people from regulatory organisations to check that the study is being carried out correctly. All will have a duty of confidentiality to you as a research participant, and we will do our best to meet this duty.

Your data may be passed to other organisations (possibly in other countries where the data protection standards and laws are different to the UK) to monitor the safety of the treatment that you are receiving, or to review results of analysis as part of the study. This data will have your name and other identifiable data removed.

What will happen if I don't want to carry on with the study?

If you withdraw consent from further study treatment, information will still be collected about you, unless you request otherwise. If you withdraw consent for further data collection your data will remain on file, and will be included in the final study analysis

The ATLANTIS Study Team may be required to collect some limited information about you regarding side effects you may have experienced as a result of taking part in the study. This will only be collected if required by the Regulatory Authorities.

Involvement of the general practitioner/family doctor:

Your GP will be kept informed of your participation in this study and we will share information you give us with your GP, if it is important to your care

What will happen to the results of the research study?

When the study is complete the results will be published in a medical journal and presented at a scientific conference. The data will be anonymous and none of the participants involved in the study will be identified in any report or

publication. The results of the study will be available to you.

Contact for further information

You are encouraged to ask any questions you wish, before, during, or after your treatment. If you have any questions about the study, please speak to your researcher or your GP, who will be able to provide you with up to date information about the drug involved.

If you are interested in taking part, please return the reply slip provided with the invitation letter or contact the research team using the details provided. If, after talking to the researcher, you decide you would like to take part, you will be asked to read and sign the consent form. You will be given a copy of this information sheet and the consent form to keep. A copy of the consent form will be included in your patient notes, one will be filed with the study records, and one will be sent to the CTRU.

Participant ID:	Initials:
Date of Birth:	NHS number:
EudraCT Number: 2019-000324-17<br><br>ISRCTN: [48075063]	Principal Investigator:

ATLANTIS
Investigating amitriptyline as second-line treatment for IBS

PARTICIPANT CONSENT FORM

Please initial each box

1. I confirm that I have read and understand the information sheet dated 30 June 2021 (version 6.0) for the above study. I have had the opportunity to consider the information, ask questions, and have had these answered satisfactorily. ☐

2. I understand that my participation in this study is voluntary and that I am free to withdraw at any time without my medical care or legal rights being affected. I understand that even if I withdraw from the above study, the data and samples collected from me will be used in analysing the results of the study and, in some cases, further information about any unwanted effects of my treatment may need to be collected by the study team. ☐

3. I agree to my data (including identifiable data and contact details) being collected and used by the Clinical Trial Research Unit (CTRU) and [INSERT RECRUITMENT HUB]. I understand they will hold this data confidentially and securely ☐

4. I give permission to allow the researcher from [INSERT RECRUITMENT HUB] and CTRU to contact me at various time points throughout the study via telephone, text message, email, or post. ☐

5. I give permission to allow the CTRU to contact me with information about what treatment (amitriptyline or placebo) I have received and also the trial results at the end of the study. ☐

6. I understand that relevant sections of my medical records and data collected in the study, may be looked at by authorised individuals from the research study team, from regulatory authorities, and from the study Sponsor, where it is relevant to my taking part in the study. I give permission for these individuals to have access to my records. This may also include anonymised data being sent outside of the EU. ☐

Participant ID:	Initials:
Date of Birth:	NHS number:
EudraCT Number: 2019-000324-17<br>ISRCTN: [48075063]	Principal Investigator:

7. I agree to my identifiable data being collected and used by Leeds Teaching Hospitals Trust (LTHT) pharmacy for the delivery of study drug.

N/A

8. **To be completed only if you are a woman who is able to have children** *(if you are a woman unable to have children, please initial the N/A box)***:** I agree for the CTRU to collect information on the outcome of any pregnancy that is recorded whilst I am in the study.

9. I agree for the CTRU to check my address with my GP.

10. I agree for my personal details (which will include my initials, date of birth, and NHS number) to be shared with providers of Electronic Health Records so that information about my healthcare use can be obtained, for future research linked to the Atlantis study, or for other research projects, subject to ethical approval

11. I agree to a copy of this Consent Form being sent to the CTRU.

12. I agree to my GP being informed of my participation in this study and being provided with a copy of this consent form.

13. I agree to take part in the study.

Optional:

Even if you agree to take part in this study, you do not have to agree to this section

Please initial

I agree, if selected, to be approached for an interview to talk about my experience in this study.

My preferred questionnaires (please circle one option):

Online **Paper**

Participant ID:	Initials:
Date of Birth:	NHS number:
EudraCT Number: 2019-000324-17<br>ISRCTN: [48075063]	Principal Investigator:

Patient:

Signature…………………………………………………………………………………

Name (block capitals)……………………………………………….………………………

Date…………………………………………………..…………………………………….

Investigator:

I have explained the study to the above named patient and I have given the patient the opportunity to discuss with a GP if they wish. The patient has indicated his/her willingness to participate.

Signature……………………………………..……………………………………

Name (block capitals)……………………………………………….……………………

Date…………………………………………………..……………………………………

(If used)Translator:

Signature……………………………………..……………………………………

Name (block capitals)……………………………………………….……………………

Date…………………………………………………..……………………………………

(1 copy for patient; 1 for the CTRU; 1 held in patient notes, original stored in Investigator Site File)
