# Supplementary material for: Amitriptyline at low-dose and titrated for irritable bowel syndrome as second-line treatment (The ATLANTIS trial): protocol for a randomised double-blind placebo-controlled trial in primary care
Source: Trials. 2022 Jul 8;23:552. doi: 10.1186/s13063-022-06492-6 (PMC9264306; doi:10.1186/s13063-022-06492-6)
Supplement: Supplementary file 3 — Additional file 3. Outcome measures and methods of data collection. [file 13063_2022_6492_MOESM3_ESM.docx]

## **Additional File 2 - Outcome measures and methods of data collection**

## **Primary Objective and Endpoint**

| **Objective** | **Endpoint** | **Method of data collection** |
| --- | --- | --- |
| What is the effect of amitriptyline, compared with placebo, on global symptoms of IBS at 6 months, measured via a widely used, validated, patient-reported, outcome measure? | IBS-SSS score at 6 months. | Questionnaire. |

## **Secondary Objectives and Endpoints**

| **Objective** | **Endpoint** | **Method of data collection** |
| --- | --- | --- |
| 1. What is the effect of amitriptyline, compared with placebo, on global symptoms of IBS at 3 and 12 months? | IBS-SSS score at 3 and 12 months. | Questionnaire. |
| 1. What is the effect of amitriptyline, compared with placebo, on relief of IBS symptoms at 3, 6 and 12 months? | Subjective Global Assessment (SGA) of relief of IBS symptoms as a dichotomous measure at 3, 6, and 12 months. | Questionnaire. |
| 1. What is the effect of amitriptyline, compared with placebo, on patient reported relief of IBS symptoms, measured weekly? | Binary response to  “Have you had adequate relief of your IBS symptoms?” asked electronically, or via a paper based diary. Participants will be sent a weekly text reminder from CTRU to complete the assessment. | Weekly question/ patient diary. |
| 1. What is the effect of amitriptyline, compared with placebo, on IBS-associated somatic symptoms at 6 months? | Patient Health Questionnaire 12 (PHQ-12) score at 6 months. | Questionnaire. |
| 1. What is the effect of amitriptyline, compared with placebo, on anxiety scores at 3, 6 and 12 months? | Hospital Anxiety and Depression Scale (HADS –  anxiety score) at 3, 6, and 12 months. | Questionnaire. |
| 1. What is the effect of amitriptyline, compared with placebo, on depression scores at 3, 6 and 12 months? | Hospital Anxiety and Depression Scale (HADS –  depression score) at 3, 6, and 12 months. | Questionnaire. |
| 1. What is the effect of amitriptyline, compared with placebo, on ability to work and participate in other activities at 3, 6 and 12 months? | Work and Social Adjustment Scale (WSAS) total score  at 3, 6 and 12 months. | Questionnaire. |
| 1. What is the acceptability of treatment with amitriptyline at 6 months, compared with placebo? | Patient-reported choice to continue active trial  medication post-6 months. Question asked by research nurse/CSO: “On balance do you find this medication acceptable to take and would you want to keep taking it”. | Nurse/CSO-completed questionnaire. |
| 1. What is the adherence to therapy with amitriptyline at 3 weeks, 3, 6, 9 and 12 months compared with placebo? | Question asked by research nurse/CSO: “Since you  were last asked, which of the options best describes  how often you have taken at least one tablet of the  trial medication daily?”   1. Every day or nearly every day 2. Half of the days or more than half the days 3. Less than half of the days 4. None or nearly none of the days   At 3 weeks, 3, 6, 9, and 12 months. | Nurse/CSO-completed questionnaire. |
| 1. What is the tolerability of amitriptyline, compared with placebo, in terms of adverse events (AEs) at 3, 6 and 12 months? | Validated Antidepressant Side- Effect Checklist (ASEC)  at 3, 6, and 12 months. | Questionnaire. |

## **Cost-effectiveness Objectives and Endpoints**

| **Objective** | **Endpoint** | **Method of data collection** |
| --- | --- | --- |
| 1. What is the effect of amitriptyline, compared with placebo, on self-reported health care use at 3, 6 and 12 months? | Cost of health care resource use  including primary, community, and  social care, admitted patient care, outpatient care (specialist visits and diagnostic investigations), cost of prescribed medications for IBS, and referral to secondary care at 3, 6, and 12 months. | Questionnaire. |
| 1. What is the effect of amitriptyline, compared with placebo, on health-related quality of life at 3, 6 and 12 months? | EQ-5D-3L time trade-off (TTO) summary score, EQ-5D-3L Visual Analogue Scale at 3, 6, and 12 months. | Questionnaire. |
| 1. What is the cost-effectiveness of amitriptyline, compared with placebo, at 6 months and 12 months? | Incremental cost-effectiveness ratio expressed in terms of incremental cost per quality adjusted life year (QALY) at 6 and 12 months. | Questionnaire. |

## **Nested Qualitative Study Objectives and Endpoints**

| **Objective** | **Endpoint** | **Data required & how is it being collected?** |
| --- | --- | --- |
| 1. What are patients’ and GPs’ experiences of treatments and participating in the trial, and how can these inform our understanding of the quantitative results and future implementation efforts? | Themes from analysis of qualitative interviews with patients and GPs. | Qualitative interviews with patients and GPs. |
| 1. To identify factors that facilitate or impede prescribing of, acceptability of, and adherence to, low-dose amitriptyline in this patient group. | Thematic analysis. | Qualitative interviews with GPs and patients. |
| 1. To identify patients’ and GPs’ perspectives on the broader impact of the trial, including any unanticipated effects not captured by the quantitative measures. | Thematic analysis. | Qualitative interviews with GPs and patients. |
| 1. To explore psychosocial and contextual factors that might shape wider use of amitriptyline for IBS. | Thematic analysis. | Qualitative interviews with GPs and patients. |

**The IBS Severity Scoring System (IBS-SSS)** is widely used in trials of medical therapies in IBS [1]. It is a 5-item self-administered questionnaire measuring presence, severity, and frequency of abdominal pain, presence and severity of abdominal distension/tightness, satisfaction with bowel habit, and degree to which IBS symptoms are affecting, or interfering with, the person’s life in general. The maximum score is 500 points: <75 points indicates symptoms that are felt to be in remission, with normal bowel function; 75-174 points indicates mild IBS symptoms; 175-299 points moderate IBS; and 300-500 points severe IBS.

**The Subjective global assessment (SGA) of relief of IBS symptoms** is frequently used in treatment trials in IBS to identify responders to therapy. Participants rate their relief from IBS symptoms on a scale of 1 to 5 ranging from "completely relieved" to "worse". Scores are dichotomised so that those scoring from 1-3 are considered responders and those 4-5 non-responders.

**The Patient Health Questionnaire 12** (**PHQ-12)** comprises 12 somatic symptoms from the full patient health questionnaire-15 [2]. Each symptom is scored from 0 ("not bothered at all") to 2 ("bothered a lot"). Higher scores indicate the presence of somatoform-type behaviour, which is a measure of psychological health.

**The Hospital Anxiety and Depression Scale (HADS)** is a well-validated, commonly used, self-report instrument for detecting anxiety and depression in people with medical illnesses [3]. It consists of a total of seven items measuring anxiety, and seven measuring depression, scored from 0 to 3, with a total score of 21 for each. Higher scores indicate more severe anxiety or depression.

**The Work and Social Adjustment Scale (WSAS)** measures the effect of chronic diseases on peoples’ ability to work and manage at home, and participate in social or private leisure activities and relationships [4]. The WSAS has been shown to be sensitive to change in IBS trials. It has five aspects scored from 0 (not affected) to 8 (severely affected), with a total possible score of 40.

**The EQ-5D-3L** is the most frequently used measure for generating QALYs [5]. It has been demonstrated to be appropriate in patients with IBS .

**Acceptability of treatment** will be measured by participant self-report, as well as the decision to continue trial medication beyond 6 months. Participants will be asked: “On balance do you find this medication acceptable to take and would you want to keep taking it”.

**Adherence** to therapy will be measured by the research nurse/CSO during the planned phone calls at 3 weeks, 3 months, 6 months, 9 months, and 12 months (as applicable). Participants will be asked: “Since you were last asked, which of the options best describes how often you have taken at least one tablet of the trial medication daily?”

- Every day or nearly every day
- More than half of the days
- Less than half of the days
- None or nearly none of the days

**AEs** will be collected via a validated self-completed questionnaire, the ASEC, which consists of 21 potential AEs rated on a scale of 0 (absent) to 3 (severe), and also asks the individual whether they deemed the AE to be treatment-related [6]. This has been shown to demonstrate good agreeement with a psychiatrist’s rating of the occurrence of treatment-related AEs with antidepressants[6]. All reported AEs will be assessed with respect to seriousness, relationship to trial medication (suspected or not suspected), and expectedness (expected or unexpected, for serious AEs).

**Health care use, use of other medications for IBS, and need for referral to secondary care** will be self-reported by the participant via a resource use questionnaire, using a 3-month recall period. If the participant consents and opts to continue the trial up to the 12-month follow-up time point, then the recall period will be extended to 6 months. This will collect data concerning all resource use and medications in the community, in primary and secondary care, social care, hospitalisations, outpatient specialist visits, and diagnostic investigations. Because of the societal perspective, the questionnaire also includes questions on out-of-pocket expenses, employment status, and days lost due to illness.

**Cost effectiveness derivations** Unit costs for health service resources will be obtained from national sources (Personal Social Services Research Unit [PSSRU]; NHS Reference Costs and British National Formulary for medicines).

Societal costs will be calculated by adding healthcare costs to the costs of lost production, based on self-reported days off work, combined with wage rates, and other reported private costs related to IBS. The intervention cost will include blood tests, drug prescriptions, and GP medication reviews.

**REFERENCES**

1 Francis CY, Morris J, Whorwell PJ. The irritable bowel severity scoring system: A simple method of monitoring irritable bowel syndrome and its progress. Aliment Pharmacol Ther 1997;**11**:395-402.

2 Spiller RC, Humes DJ, Campbell E, Hastings M, Neal KR, Dukes GE*, et al.* The Patient Health Questionnaire 12 Somatic Symptom scale as a predictor of symptom severity and consulting behaviour in patients with irritable bowel syndrome and symptomatic diverticular disease. Aliment Pharmacol Ther 2010;**32**:811-20.

3 Zigmond AS, Snaith RP. The hospital anxiety and depression scale. Acta Psychiatr Scand 1983;**67**:361-70.

4 Mundt JC, Marks IM, Shear MK, Greist JH. The Work and Social Adjustment Scale: A simple measure of impairment in functioning. Br J Psychiatry 2002;**180**:461-4.

5 Group E. EuroQol--a new facility for the measurement of health-related quality of life. Health Policy 1990;**16**:199-208.

6 Uher R, Farmer A, Henigsberg N, Rietschel M, Mors O, Maier W*, et al.* Adverse reactions to antidepressants. Br J Psychiatry 2009;**195**:202-10.
