# Supplementary material for: Amitriptyline at low-dose and titrated for irritable bowel syndrome as second-line treatment (The ATLANTIS trial): protocol for a randomised double-blind placebo-controlled trial in primary care
Source: Trials. 2022 Jul 8;23:552. doi: 10.1186/s13063-022-06492-6 (PMC9264306; doi:10.1186/s13063-022-06492-6)
Supplement: Supplementary file 4 — Additional file 4. REC Approval Letter. [file 13063_2022_6492_MOESM4_ESM.pdf]

Yorkshire & The Humber - Sheffield Research Ethics Committee

NHSBT Newcastle Blood Donor Centre
Holland Drive
Newcastle upon Tyne
NE2 4NQ

Tel: 0207 104 8082

Please note: This is the favourable opinion of the REC only and does not allow you to start your study at NHS sites in England until you receive HRA Approval

2 August 2019

Professor Alexander Ford
Leeds Institute of Medical Research
Room 7.09 Worsley Building
Leeds LS2 9JT

Dear Professor Ford

**Study title:**	**Amitriptyline at Low-dose and Titrated for Irritable Bowel Syndrome as Second-line Treatment (The ATLANTIS study): A Double-blind Placebo-controlled Trial**
**REC reference:**	**19/YH/0150**
**Protocol number:**	**GA18/118305**
**EudraCT number:**	**2019-000324-17**
**IRAS project ID:**	**252282**

Thank you for your response of 19 July 2019, responding to the Committee's request for further information on the above research and submitting revised documentation.

The further information was considered in correspondence by a Sub-Committee of the REC. A list of the Sub-Committee members is attached.

Confirmation of ethical opinion

The Committee has considered your response and the letter from the chair of the TSC and sought further advice from the REC pharmacist. The Committee noted that the IMP should be used as recommended in the BNF.

On behalf of the Committee, I am pleased to confirm a **Favourable** ethical opinion for the above research on the basis described in the application form, protocol and supporting documentation as revised, subject to the conditions specified below.

Conditions of the favourable opinion

The REC favourable opinion is subject to the following conditions being met prior to the start of the study.

<u>Confirmation of Capacity and Capability (in England, Northern Ireland and Wales) or NHS management permission (in Scotland) should be sought from all NHS organisations</u>

involved in the study in accordance with NHS research governance arrangements. Each NHS organisation must confirm through the signing of agreements and/or other documents that it has given permission for the research to proceed (except where explicitly specified otherwise).

Guidance on applying for HRA and HCRW Approval (England and Wales)/ NHS permission for research is available in the Integrated Research Application System.

For non-NHS sites, site management permission should be obtained in accordance with the procedures of the relevant host organisation.

Sponsors are not required to notify the Committee of management permissions from host organisations

Registration of Clinical Trials

It is a condition of the REC favourable opinion that all clinical trials are registered on a publicly accessible database. For this purpose, clinical trials are defined as the first four project categories in IRAS project filter question 2. For clinical trials of investigational medicinal products (CTIMPs), other than adult phase I trials, registration is a legal requirement.

Registration should take place as early as possible and within six weeks of recruiting the first research participant at the latest. Failure to register is a breach of these approval conditions, unless a deferral has been agreed by or on behalf of the Research Ethics Committee (see here for more information on requesting a deferral: https://www.hra.nhs.uk/planning-and-improving-research/research-planning/research-registration-research-project-identifiers/

As set out in the UK Policy Framework, research sponsors are responsible for making information about research publicly available before it starts e.g. by registering the research project on a publicly accessible register. Further guidance on registration is available at: https://www.hra.nhs.uk/planning-and-improving-research/research-planning/transparency-responsibilities/

You should notify the REC of the registration details. We will audit these as part of the annual progress reporting process.

Publication of Your Research Summary

We will publish your research summary for the above study on the research summaries section of our website, together with your contact details, no earlier than three months from the date of this favourable opinion letter. Should you wish to provide a substitute contact point, make a request to defer, or require further information, please visit: https://www.hra.nhs.uk/planning-and-improving-research/application-summaries/research-summaries/

Clinical trial authorisation must be obtained from the Medicines and Healthcare products Regulatory Agency (MHRA).

The sponsor is asked to provide the Committee with a copy of the notice from the MHRA, either confirming clinical trial authorisation or giving grounds for non-acceptance, as soon as this is available.

It is the responsibility of the sponsor to ensure that all the conditions are complied with before the start of the study or its initiation at a particular site (as applicable).

After ethical review:

Reporting requirements

The attached document "After ethical review – guidance for researchers" gives detailed guidance on reporting requirements for studies with a favourable opinion, including:

- Notifying substantial amendments
- Adding new sites and investigators
- Notification of serious breaches of the protocol
- Progress and safety reports
- Notifying the end of the study, including early termination of the study
- Final report.

The latest guidance on these topics can be found at https://www.hra.nhs.uk/approvals-amendments/managing-your-approval/.

Ethical review of research sites

NHS/HSC sites

The favourable opinion applies to all NHS/HSC sites listed in the application subject to confirmation of Capacity and Capability (in England, Northern Ireland and Wales) or management permission (in Scotland) being obtained from the NHS/HSC R&D office prior to the start of the study (see "Conditions of the favourable opinion" below).

Non-NHS/HSC sites

I am pleased to confirm that the favourable opinion applies to any non-NHS/HSC sites listed in the application, subject to site management permission being obtained prior to the start of the study at the site.

Approved documents

The final list of documents reviewed and approved by the Committee is as follows:

Document	*Version*	*Date*
Copies of advertisement materials for research participants [ATLANTIS clinic poster_advert]	1.0	05 April 2019
Covering letter on headed paper [ATLANTIS REC Cover Letter]		11 April 2019
Evidence of Sponsor insurance or indemnity (non NHS Sponsors only) [Clinical Trials Insurance cover letter]		27 September 2018
GP/consultant information sheets or letters [ATLANTIS GP Letter]	1.0	05 April 2019
Investigator's brochure / IMP Dossier [ATLANTIS IMPD]	1.0	21 March 2019
IRAS Application Form [IRAS_Form_15062019]		15 June 2019
Letter from funder [16.162.01 Ford- Agree to fund letter]		08 February 2018
Letter from sponsor [Sponsor Authorisation to Apply for HRA submission]		09 April 2019
Letters of invitation to participant [ATLANTIS Invitation Letter]	1.0	05 April 2019
Non-validated questionnaire [ATLANTIS SGA Questionnaire]	1.0	05 April 2019
Non-validated questionnaire [ATLANTIS Socio demographic questionnaire]	1.0	05 April 2019
Non-validated questionnaire [ATLANTIS Previous Anxiety / Depression questionnaire]	1.0	05 April 2019
Non-validated questionnaire [ATLANTIS PHQ-12]	2.0	14 June 2019
Non-validated questionnaire [ATLANTIS WSAS Questionnaire]	2.0	14 June 2019
Non-validated questionnaire [ATLANTIS Health Resource Use baseline]	2.0	14 June 2019

Non-validated questionnaire [ATLANTIS Health Resource Use month 6]	2.0	14 June 2019
Non-validated questionnaire [ATLANTIS Health Resource Use month 3]	2.0	14 June 2019
Non-validated questionnaire [ATLANTIS Health Resource Use month 12]	2.0	14 June 2019
Other [ATLANTIS Participant Dose Adjustment Information]	1.0	05 April 2019
Other [ATLANTIS Participant Symptom paper diary (0-3 months)]	1.0	05 April 2019
Other [ATLANTIS Participant Symptom paper diary (3-6 months)]	1.0	05 April 2019
Other [ATLANTIS Weekly participant symptom question, electronic]	1.0	05 April 2019
Other [ATLANTIS Weekly text reminder paper diary]	1.0	05 April 2019
Other [ATLANTIS Initial Email Text OnlineQ]	1.0	05 April 2019
Other [ATLANTIS Initial Email Text PostalQ_]	1.0	05 April 2019
Other [ATLANTIS Reminder Email Text OnlineQ]	1.0	05 April 2019
Other [ATLANTIS Reminder Email Text PostalQ]	1.0	05 April 2019
Other [IBS dietary advice sheet (BDA Food Facts Sheet)]	n/a	01 January 2019
Other [ATLANTIS_Qual Invitation Letter for Patients]	1.0	05 April 2019
Other [ATLANTIS Qual Patient Interview Topic Guide]	1.0	05 April 2019
Other [ATLANTIS Qual Invitation Email for GPs]	1.0	05 April 2019
Other [ATLANTIS Qual GP Interview topic Guide]	1.0	05 April 2019
Other [Portfolio Application Form (PAF)]	1.0	25 March 2019
Other [ATLANTIS trial entry text _clean version]	2.0	14 June 2019
Other [ATLANTIS trial entry text]	2.0	14 June 2019
Other [ATLANTIS Qual Patient Interview Information Sheet]	2.0	14 June 2019
Other [ATLANTIS Qual Patient Interview Consent Form]	2.0	14 June 2019
Other [ATLANTIS Qual GP Interview Information Sheet]	2.0	14 June 2019
Other [ATLANTIS Qual GP Interview consent form]	2.0	14 June 2019
Other [ATLANTIS Website Content]	1.0	14 June 2019
Other [ATLANTIS Participant Pack Baseline]	1.0	14 June 2019
Other [ATLANTIS Participant Pack Month 3]	1.0	14 June 2019
Other [ATLANTIS Participant Pack Month 6]	1.0	14 June 2019
Other [ATLANTIS Participant Pack Month 12]	1.0	14 June 2019
Other [ATLANTIS REC Cover Letter Response to Provisional Opinion]	1.0	14 June 2019
Other [ATLANTIS Qual GP Interview Consent From TRACKED]	2.0	14 June 2019
Other [ATLANTIS Qual GP interview information sheet_TRACKED]	2.0	14 June 2019
Other [ATLANTIS Qual Patient Interview Consent Form_TRACKED]	2.0	14 June 2019
Other [ATLANTIS Qual Patient Interview Information Sheet_TRACKED]	2.0	14 June 2019
Other [ATLANTIS_REC Cover Letter_incomplete response]	1.0	19 July 2019
Other [TSC letter to Ethics Committee]	N/A	18 July 2019
Participant information sheet (PIS) [ATLANTIS Patient Information Sheet and Informed Consent Form (tracked version)]	3.0	19 July 2019
Participant information sheet (PIS) [ATLANTIS Patient Information Sheet and Informed Consent Form (clean version)]	3.0	19 July 2019
Referee's report or other scientific critique report [IBS Network Peer Review]		29 March 2017
Referee's report or other scientific critique report [PCSG peer review]		24 March 2017
Referee's report or other scientific critique report [BSG peer review]		24 March 2017
Research protocol or project proposal [ATLANTIS Protocol]	1.0	05 April 2019
Sample diary card/patient card [ATLANTIS Participant ID Card]	1.0	05 April 2019
Summary CV for Chief Investigator (CI) [Alex Ford Short CV]		05 April 2019

Summary of product characteristics (SmPC) [Teva NL Amitriptyline 10mg ENGLISH SPC]		20 December 2018
Summary, synopsis or diagram (flowchart) of protocol in non-technical language [ATLANTIS Protocol Summary]	1.0	05 April 2019
Validated questionnaire [IBS-SSS]		
Validated questionnaire [HADS]		
Validated questionnaire [EQ-5D-3L]		
Validated questionnaire [ROME IV Diagnostic Criteria]		
Validated questionnaire [ATLANTIS ASEC (Antidepressant Side-Effect Checklist questionnaire)_modified]	V1.0	14 June 2019

Statement of compliance

The Committee is constituted in accordance with the Governance Arrangements for Research Ethics Committees and complies fully with the Standard Operating Procedures for Research Ethics Committees in the UK.

User Feedback

The Health Research Authority is continually striving to provide a high quality service to all applicants and sponsors. You are invited to give your view of the service you have received and the application procedure. If you wish to make your views known please use the feedback form available on the HRA website: http://www.hra.nhs.uk/about-the-hra/governance/quality-assurance/

HRA Learning

We are pleased to welcome researchers and research staff to our HRA Learning Events and online learning opportunities– see details at: https://www.hra.nhs.uk/planning-and-improving-research/learning/

**19/YH/0150**	**Please quote this number on all correspondence**

With the Committee's best wishes for the success of this project.

Yours sincerely
pp

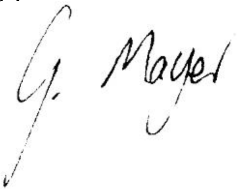

Professor Basil Sharrack
Chair

Email:nrescommittee.yorkandhumber-sheffield@nhs.net

Enclosures: List of names and professions of members who were present at the meeting and those who submitted written comments

'After ethical review – guidance for researchers'

Copy to: Dr Heather Cook – Research Dept, University of Leeds

Lead Nation - England

Yorkshire & The Humber - Sheffield Research Ethics Committee

Attendance at Sub-Committee of the REC meeting on 1 August 2019 in correspondence

Committee Members:

Name	*Profession*	*Present*	*Notes*
Professor Basil Sharrack (Chair)	Consultant Neurologist	Yes	
Mrs Aikaterini Staikoura-Wilson	Pharmacist	Yes	
Dr Liz Williams	Senior Lecturer in Human Nutrition	Yes	

Also in attendance:

Name	*Position (or reason for attending)*
Ms Gillian Mayer	Approvals Officer
